# Comparing brass mesh to tissue equivalent bolus materials for volumetric modulated arc therapy chest wall irradiation

**DOI:** 10.1002/acm2.14054

**Published:** 2023-06-07

**Authors:** Timothy D. Keiper, Kelly Kisling, Patricia Hua, Ryan P. Manger

**Affiliations:** ^1^ Department of Radiation Medicine and Applied Sciences Moores Cancer Center University of California San Diego La Jolla California USA; ^2^ California Protons Cancer Therapy Center San Diego California USA

**Keywords:** brass bolus, brass mesh, VMAT breast

## Abstract

**Purpose:**

To compare the superficial dose when using brass mesh bolus (BMB), no bolus, or 3 mm tissue‐equivalent bolus with a pseudo‐flash volumetric modulated arc therapy (VMAT) breast treatment planning technique.

**Methods:**

Two different beam arrangements for right‐sided irradiation and one beam arrangement for bilateral irradiation were planned on an inhomogeneous thorax phantom in accordance with our clinical practice for VMAT postmastectomy radiotherapy (PMRT). Plans were optimized using pseudo‐flash and representative critical organ optimization structures were used to shape the dose. Plans were delivered without bolus, with 3 mm tissue‐equivalent bolus (TEB), or with one‐layer BMB. Optically stimulated luminescence dosimeter (OSLD) and radiochromic film measurements were taken and analyzed to determine the superficial dose in each case and the relative enhancement from the no bolus delivery.

**Results:**

Superficial dose measured with OSLDs was found to be 76.4 ± 4.5%, 103.0 ± 6.1%, and 98.1 ± 5.8% of prescription for no physical bolus (NB), TEB, and BMB, respectively. Superficial dose was observed to increase from lateral to medial points when measured with film. However, the relative increase in superficial dose from NB was consistent across the profile with an increase of 43 ± 2.1% and 34 ± 3.3% of prescription for TEB and BMB, respectively. The results are in good agreement with expectations from the literature and the experience with tangential radiotherapy.

**Conclusion:**

Three millimeter TEB and one‐layer BMB were shown to provide similar enhancement to the superficial dose compared to delivery without bolus. BMB, which does not significantly affect dose at depth and is more conformal to the patient surface, is an acceptable alternative to 3 mm TEB for chest wall PMRT patients treated with pseudo‐flash PMRT.

## 1 INTRODUCTION

The American Cancer Society states that breast cancer accounts for about 30% of all new female cancers each year with a projection of more than 325 000 new diagnoses in 2022.^1^ 3D conformal radiotherapy (3DCRT) has historically been the preferred method of treatment delivery due to the advantageous simplicity of the treatment site. However, recent publications for volumetric modulated arc therapy (VMAT) postmastectomy radiotherapy (PMRT) have reported on the low complication rates for treatment with VMAT delivery.^2^, ^3^ Given the ability to preferentially spare critical structures in PMRT,^4^ VMAT treatment planning and delivery for chest wall radiation is expected to increase in clinics worldwide.

Flash has been used for many years to account for setup uncertainty and treatment volume changes in 3DCRT. In analog, pseudo‐flash planning strategies for VMAT delivery have been developed and described in the literature.^5^, ^6^, ^7^ From these references, the PTV should be extended at least 5 mm from the chest wall and a virtual bolus (v‐bolus) which extends beyond the extended PTV should be added as a planning structure. The authors of these works suggest that when variation from the planned position of about 10 mm is observed the patient should be considered for replanning.

In PMRT patients, clinicians usually intend to treat the skin and typically prescribe the use of physical bolus (p‐bolus) to increase the superficial dose in the presence and absence of breast reconstruction.^8^ Regimens for p‐bolus can become complicated and cause confusion among the staff, particularly for busy clinics. While the use of p‐bolus in PMRT is itself a point of controversy in the community,^9^, ^10^ understanding the available tools to control the superficial dose is imperative to providing the best patient experience and outcomes.

Manger et al. report clinically equivalent superficial dose enhancement to 75%–110% for brass mesh p‐bolus (BMB) and 85%–109% for 5 mm tissue‐equivalent p‐bolus (TEB) for tangential PMRT.^11^ Al‐Rahbi et al. characterized the superficial dose of BMB as compared to 5 and 10 mm of TEB and no p‐bolus (NB) scenarios.^12^ They report that while BMB increases the superficial dose, it is not as significant as 5 and 10 mm of TEB. They also note that the superficial dose enhancement from BMB primarily comes from backscatter of the exit dose. Al‐Rahbi et al. confirm the report from Manger et al. that BMB did not have a significant impact on dose at depth. Fiedler et al. investigated the skin dose enhancements of BMB compared to 3 mm of TEB with 3DCRT, electronic compensator, field‐in‐field, and VMAT techniques.^13^ They report that one layer of BMB produces equivalent enhancement to 3 mm of TEB with 88.4% and 93.5% skin dose for BMB and TEB, respectively. However, the VMAT plan in their work was limited to two‐180‐degree arcs and were optimized without v‐bolus or flash. Monajemi et al. reported the dose enhancement for different p‐bolus regimens using pseudo‐flash VMAT to 83% for BMB and 96% for 3 mm TEB using a custom phantom based on a specific patient.^14^ This work included the pseudo‐flash VMAT component but lacks a variety of field arrangements to confirm the robust process of this planning strategy.

In this paper, we investigate the use of BMB compared to 3 mm of TEB in conjunction with pseudo‐flash VMAT planning based on our clinical needs. 3 mm bolus is commonly prescribed by our clinicians for about 50% of fractions until the desired skin reaction is clinically observed for VMAT PMRT. In the previously described publications, BMB has been shown to be dosimetrically equivalent to 3 mm of TEB while not having a significant impact on the dose delivery at depth. The impact of 3 mm TEB on the dose profile consumes clinical resources by requiring replanning to account for this difference. Additionally, BMB has superior topographic conformality when compared to TEB options and can be advantageous for patient setup and minimization of dosimetric uncertainty introduced by airgaps present when using TEB for challenging chest wall cases. We studied three total beam arrangements, two unilateral arrangements, and one bilateral arrangement, to build on the report by Monajemi et al. and to demonstrate the robust approach of pseudo‐flash VMAT optimization using BMB to increase the superficial dose for post‐mastectomy patients treated with radiation therapy. The setup presented here uses a common inhomogeneous phantom for planning and delivery accessible to most practices with the goal of providing valuable guidance for clinics looking to verify their individual processes.

## 2 METHODS

**FIGURE 1:**
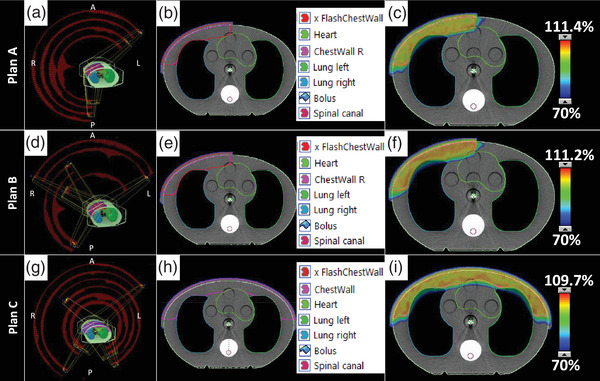
VMAT planning process showing (A, D, G) the arc angles and control points, (B, E, H) the structure delineation used in optimization, and (C, F, I) the dose color wash to the 70% coverage level showing clinically acceptable organ sparing and dose conformality for (top) Plan A, (middle) Plan B, and (bottom) Plan C arrangements.

Chest wall VMAT treatment plans with a prescription to 200 cGy per fraction were generated using Eclipse 16.1 (Varian Medical Systems, Palo Alto, CA). Photon Optimizer was used for VMAT optimization, and Analytical Anisotropic Algorithm was used for volume dose calculation. Treatment plans were generated using the flattened 6 MV beam energy configured on a TrueBeam linear accelerator (Varian Medical Systems, Palo Alto, CA). A planning CT of a CIRS Thorax phantom (CIRS, Inc., Norfolk, VA) was acquired on a GE Discovery CT Simulator (GE Discovery, GE Healthcare, Chicago, IL). The planning CT was acquired without bolus or measurement devices to mimic our standard operating procedure for patient CT simulation. Structure sets were created for unilateral, right‐sided irradiation, and bilateral irradiation. This work focuses on the superficial dose and therefore did not focus on nodal coverage or include simultaneous integrated boost nodal volumes. The lung structure of the phantom was contoured and structures representing the heart, spinal cord, and midline organs at risk (OARs) were drawn to define realistic optimization objectives. A chest wall structure was delineated along the phantom from the mid‐coronal plane to either the midline for the right‐sided plans or extending across the entire anterior of the phantom in the bilateral case. It extended about 2 cm in depth from the phantom surface and was cropped from OARs without margin. Following guidelines based off the pseudo‐flash technique in the literature,^15^ the chest wall PTV was expanded 5 mm anterior and lateral outside of the body. A 7 mm water‐equivalent v‐bolus was inserted in the region of the PTV for optimization based on our clinical practice. Our clinical practice differs from the literature based on the preference to minimize the dosimetric impact when the pseudo‐flash plan is recalculated without bolus. Three total VMAT field arrangements were investigated: 4‐arc (Plan A) and 5‐arc (Plan B) geometries for the unilateral site and a 6‐arc (Plan C) geometry for the bilateral site. Specifics of the field arrangement are shown in Table 1. Arc angles, structure delineation, and dose to the 70% level are shown in Figure 1.

**TABLE 1:** Volumetric modulated arc therapy (VMAT) field arrangement parameters.

	Arc range (°)	Collimator angle (°)	Jaw positions (X1, X2, Y1, Y2)
Plan A – 4 arc	50 – 181 181 – 50 50 – 181 181 – 50	355.0 5.0 90.0 90.0	10.0, 4.5, 9.1, 9.1 4.5, 10.0, 9.1, 9.1 4.0, 8.8, 9.6, 9.5 8.7, 4.0, 9.6, 9.5
Plan B – 5 arc	300 – 60 60 – 300 330 – 210 210 – 330 300 – 60	355.0 5.0 355.0 5.0 90.0	9.9, 4.6, 9.0, 9.1 4.0, 10.5, 9.1, 9.0 9.9, 4.6, 9.0, 9.0 4.0, 10.5, 9.0, 9.1 2.0, 8.8, 9.9, 9.2
Plan C – 6 arc bilat	50 – 215 215 – 50 155 – 310 310 – 155 155 – 215 215 – 155	350.0 10.0 355.0 5.0 90.0 90.0	12.5, 3.5, 10.5, 10.5 3.5, 12.5, 10.5, 10.5 11.0, 5.0, 10.5, 10.5 5.0, 11.0, 10.5, 10.5 10.0, 6.0, 16.5, 16.5 6.0, 10.0, 16.5, 16.5

**FIGURE 2:**
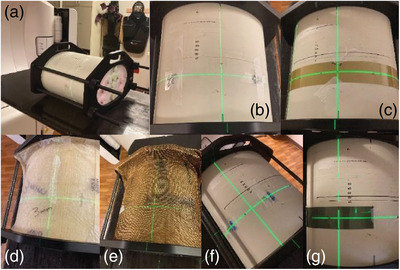
Phantom setup for delivery and measurement. (A) Phantom aligned to BBs before IGRT. (B) Phantom aligned after applying IGRT (CBCT) shifts for the bilateral case with OSLDs placed on either side of isocenter. (C) Film measurement placement for Plan C. (D) 3 mm TEB placed on the phantom for irradiation. (E) BMB placed on the phantom for irradiation. (F) OSLD Placement for unilateral measurements. (G) Film placement after irradiation of unilateral setup.

Prior to taking measurements of each plan, the phantom was aligned using a similar image‐guided protocol. After initial alignment of the phantom BBs to the lasers, image‐guidance using cone‐beam computed tomography (CBCT) was performed with the planning CT as reference. Alignment was performed using four degrees of freedom shifts and yaw rotations. The same shifts were used for all measurements for a given beam arrangement because the planning isocenter was fixed. Three p‐bolus options were investigated for each of the three plans: NB, 3 mm Superflab (RPD, Inc., Albertville, MN) TEB, or 1‐layer of BMB (RPD, Inc. Albertville, MN) covering the entire anterior aspect of the phantom (Figure 2). Optically stimulated luminescence dosimeter (OSLD; nanoDot, Landauer, Glenwood, IL) have a manufacturer‐documented measurement accuracy of ±5.5% for screened nanoDots. Calibration for OSLD measurements were conducted in our clinic following the standard protocol with irradiation in a 6 MV photon field. A linear calibration curve was generated for doses from 0 to 3 Gy. Overall uncertainty of the OSLD measurement is approximated to be ±5.9%. OSLD measurement locations were marked on the phantom for placement consistency as shown in Figure 2. OSLDs were placed on the right and left side of the sagittal laser and along the axial laser. For each setup, two sets of OSLD measurements were acquired to reduce uncertainty in OSLD readings from the inherent OSLD uncertainty and small differences in delivery. Film dosimetry was acquired using radiochromic film (Gafchromic EBT3, Ashland, Bridgewater, NJ) calibrated following standard procedure in a 6 MV photon field. The calibration curve was made for doses from 0 to 3 Gy. The uncertainty in film measurements is approximated to be ±2%. For each setup, one film measurement was taken with a 2.5 cm wide by 20 cm long film calibrated for 6 MV, oriented along the axial laser from the mid‐sagittal plane of the phantom. For the bilateral plan, film was positioned from the midline to both the right and left lateral extents.

## 3 RESULTS

OSLD readings are detailed in Table 2. Superficial dose for NB, 3 mm TEB, and BMB for the three plans was 76.4 ± 4.5%, 103.0 ± 6.1%, and 98.1 ± 5.8%, respectively, of the prescribed dose. For both Plan A and B, the lateral OSLD recorded a lower dose than the medial OSLD. The difference between the lateral and medial measurement for NB, 3 mm TEB, and BMB for Plan A was 10.5%, 4.5%, and 7%, respectively, while for Plan B, the difference was 11%, 3%, and 8%, respectively. For Plan C, OSLDs were measured at approximately the same distance from midline and there was not as much discrepancy between the NB, 3 mm TEB, and BMB cases at 2%, 2.5%, and 1.5%, respectively.

**TABLE 2:** Optically stimulated luminescence dosimeter (OSLD) measurements of superficial dose

	Bolus	Average OSLD reading (cGy)^a^	% of Rx
Plan A	No bolus 3 mm TEB BMB	151.5 205.5 194	75.8 102.8 97.0
Plan B	No bolus 3 mm TEB BMB	152 205 195	76.0 102.5 97.5
Plan C	No bolus 3 mm TEB BMB	155 207.5 199.5	77.5 103.8 99.8

^a^ Overall uncertainty in OSLD measurement is approximated to be ±5.9% of the measured value.

**FIGURE 3:**
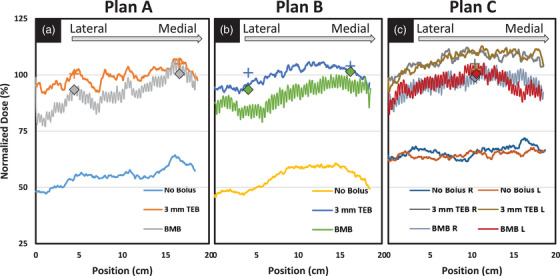
Film (line) and OSLD (markers) dosimetry for the three plan delivery types for each bolus option. (A) Plan A, (B) Plan B, and (C) Plan C deliveries show similar trends and good agreement between OSLD and film measurements.

Film and OSLD dosimetry measurements are depicted in Figure 3. OSLD measurements for the p‐bolus cases are represented by the markers and film is shown with solid lines. Film dosimetry agrees with the point measurements and demonstrates the decreasing surface dose trend from the medial to lateral aspect of the phantom. These measurements show a dependence of the position along the curvature of the phantom surface. At a given point, the average increase of the superficial dose for 3 mm TEB and BMB beyond the native dose of the NB case is 43 ± 2.1% and 34 ± 3.3%, respectively.

## 4 DISCUSSION

The results presented in this paper agree well with previously published results on tangential PMRT with p‐bolus. The VMAT dose variation along the phantom curvature was nearly identical to the superficial dose range with tangential fields published by Manger et al.^11^ Superficial dose enhancement with BMB and TEB were similar to other reported values despite the particular beam arrangement (Table 3).^13^, ^14^ Al‐Rahbi et al.^12^ did not oppose the beam in their open field measurement which we interpret as the reason they report a significantly lower surface dose compared to the other listed publications.

**TABLE 3:** Superficial dose measurements using EBT‐3 radiochromic film for one‐layer brass mesh bolus (BMB) from the literature

Author (Year)	Technique	Measurement %
Manger et al. (2016)^11^	Tangent	75%–110%
Al‐Rahbi et al. (2018)^12^	Open 10 × 10 cm^2^	56.7 ± 5.3%
Monajemi et al. (2020)^14^	VMAT, 3−215° Arcs	83%
Fiedler et al. (2021)^13^	VMAT, 2−180° Arcs	88.4 ± 5.2%

Monajemi et al.^14^ investigated the superficial dose enhancement with various p‐bolus options for pseudo‐flash VMAT for PMRT and report similar results to those in this manuscript. We differentiate our work from theirs in a few key areas. First, the optimization in their work uses 10 mm v‐bolus while ours uses only 7 mm v‐bolus. Second, we expand on their results by investigating a variety of field geometries over a larger profile for unilateral and bilateral treatments. Finally, our experimental design implements a commonly accessible thorax phantom and thus provides a valuable set of data for comparison by other clinics looking to implement pseudo‐flash VMAT PMRT with p‐bolus.

VMAT planning resulted in a decreased dose on the lateral aspect of the phantom consistent with an increased curvature of the surface. In contrast to Monajemi et al. who reported negligible variability for the central 8 cm of the treatment area,^14^ the data in this manuscript are reported for approximately 20 cm along the chest wall and shows significant variability. We believe that our measurements demonstrate similar profiles over smaller sections of the chest wall, but acknowledge that deviations may be a result of the planning process. In our measurements, the variation in surface dose is most evident at the medial and lateral aspects of the film. This variation was seen in all plans with and without p‐bolus. However, the relative dose enhancement across the profile is consistent for a given p‐bolus choice regardless of position along the chest wall. The apparent noise in the BMB film measurements is due to the construction of the bolus where individual brass sections are connected with brass rings. The effects of this variation in surface dose should wash out with minor variations in bolus placement for highly fractionated cases. Clinicians concerned with the superficial dose are encouraged to use in vivo dosimetry to confirm the dose along the curvature of the chest wall.

OSLD measurements were observed to differ significantly from the film measurements in the NB case. Accurate measurement of the superficial dose is challenging due to the rapid buildup region. OSLDs have an effective measurement depth of 0.5 mm when jacketed. In a similar study including NB superficial dose, the bare versus jacketed OSLD measurement increased from 53% to 64% of prescription.^14^ Therefore, it is not surprising that OSLDs would overestimate the superficial dose compared to film when measuring without p‐bolus. OSLDs measured under p‐bolus are in a more stable portion of the buildup region and are expected to have less uncertainty in their specific measurements and more accurately represent the dose at the nominal depth.

Measurements at depth were not in the scope of this work. Previous studies have demonstrated the value of BMB and its minimal impact on dose at depth. The literature significantly supports this claim and therefore we omitted any measurements on that topic from this study. However, we recognize the importance of fully characterizing the dosimetric impact of bolus materials and encourage clinics wishing to implement a new bolus strategy to fully evaluate the resultant dose of their process.

## 5 CONCLUSION

The use of brass mesh p‐bolus in the clinic can be beneficial for increasing skin dose to the patient without affecting dose at depth and therefore eliminating the need to replan when p‐bolus is discontinued while maintaining comparable dosimetry to the original plan. The conformality provided by BMB is advantageous for dosimetric integrity and patient setup. The superficial dose enhancement due to BMB for VMAT PMRT using the pseudo‐flash technique has been shown in this work to be equivalent to the use of 3 mm TEB. Considering the additional advantages, BMB could be preferred over 3 mm TEB and should be considered an acceptable alternative for clinical PMRT treatments.

## AUTHOR CONTRIBUTION

Timothy D. Keiper developed, carried out, and analyzed the measurements and prepared the manuscript. Kelly Kisling collected data and edited the manuscript for intellectual content. Patricia Hua assisted with the planning stages and edited the manuscript for intellectual content. Ryan P. Manger oversaw the methods and measurements, analyzed data, and prepared the manuscript.

## CONFLICT OF INTEREST STATEMENT

This investigation was reported at the 2022 AAPM Annual Meeting. Authors have no COI to declare.

## Data Availability

The data that support the findings of this study are available from the corresponding author upon reasonable request.
